# Prolonged retention of gauze sponge resulting in ileocolic fistula, a rare complication following cesarean section; case report

**DOI:** 10.1016/j.ijscr.2023.109081

**Published:** 2023-11-20

**Authors:** Willbroad Kyejo, Allyzain Ismail, Sajida Panjwani, Shabbir Adamjee, Sunil Samji, Ally Mwanga

**Affiliations:** a The Aga Khan University, East Africa Medical college, Tanzania; b Department of Anaesthesia, The Aga Khan Hospital, Dar-es-Salaam, Tanzania; c Department of Surgical Gastroenterology, Muhimbili University of Health and Allied Sciences, Dar-Es-Salaam, Tanzania

**Keywords:** Retained gauze sponge, Foreign body, Ileocolic fistula, Case report

## Abstract

**Introduction and importance:**

Retained gauze sponge is a medical legal issue with significant clinical implications with catastrophic complications. We report a case of a female who presented with chronic right iliac fossa pain only to be found to have a retained gauze sponge causing bowel fistulisation. We describe our experience on diagnostic formulation and work up and subsequent operative intervention.

**Case presentation:**

We present the case of a 37-year-old female patient who presented to the outpatient surgical department with symptoms of chronic right iliac fossa pain with a history of cesarean section 2 years prior. A computed tomography scan revealed an inflammatory mass and operative exploration revealed a retained gauze sponge causing a fistula between the terminal ileum and caecum. Underwent a right hemicolectomy with an uneventful postoperative period.

**Clinical discussion:**

Retained gauzes can lead to a spectrum of complications including fistulisation presenting with vague non-specific abdominal symptoms. The subtle presentation challenges the clinician to consider the possibility of retained foreign bodies in patient with history of abdominal surgeries. This emphasizes the importance of policies enforcing swab count as a simple retained gauze led to catastrophic complication and ultimately a right hemicolectomy.

**Conclusion:**

This case report presents a complex and instructive clinical scenario, emphasizing the challenges of diagnosing atypical presentations of retained foreign bodies, the critical importance of surgical counting protocols, and the implications for patient safety and quality of care.

## Highlights

• Importance of assessing for swab count at the end of procedure
• Clinical implications of retained foreign body
• Non-specific symptoms hence difficult to diagnose
• Patient safety protocols to prevent catastrophic complications

## 1 Introduction and importance

Foreign body retention following surgical procedures represents a rare yet potentially severe complication with significant implications for patient health and healthcare systems [1]. This phenomenon occurs when surgical items, such as sponges or instruments, are inadvertently left within a patient's body during surgery [2,3]. Such retained foreign bodies can lead to a spectrum of adverse outcomes, including infection, abscess formation, and, as illustrated in this case, the development of fistulas [4].

The pathophysiology of retained foreign bodies is rooted in the body's response to an unrecognized foreign object [5]. When a surgical item is left behind, it can incite an inflammatory response as the body attempts to encapsulate and isolate the foreign material [6,7]. Over time, this inflammatory process can lead to the formation of fistulous tracts, creating abnormal connections between different anatomical structures, such as the small intestine and the colon, as demonstrated in this case [8]. These fistulas may result in chronic symptoms, including abdominal pain, diarrhea, and fever, as well as other complications [4].

Several factors contribute to the risk of retained foreign bodies. Complex surgical procedures, high patient turnover, and emergent surgeries can increase the likelihood of errors during the counting and retrieval of surgical items [9,10]. Furthermore, human factors, such as distractions or fatigue in the operating room, can play a role in these incidents. The burden of retained foreign bodies extends beyond patient health, encompassing financial costs, increased hospital stays, legal consequences, and the emotional toll on both patients and healthcare providers [11].

This case report illuminates the multifaceted challenges and complications associated with retained foreign bodies, emphasizing the importance of early recognition, prevention strategies, and a comprehensive understanding of the pathophysiological processes involved. By delving into this complex clinical scenario, we aim to contribute to the collective knowledge surrounding the complications of foreign body retention, promote awareness among healthcare professionals for continued efforts to enhance patient safety in surgical practice by adapting strict swab count policies and practices. This paper has been reported in line with the SCARE 2020 criteria [12]. This article has been registered with the Research Registry.

## 2 Case presentation

A 37-year-old female, with an unremarkable medical history aside from a cesarean section two years prior, presented to our hospital with a perplexing constellation of symptoms. Over the preceding several months, she complained of persistent and increasingly debilitating abdominal pain localized to the right lower quadrant (RLQ). Alongside the pain, she described recurrent episodes of diarrhea and noted the presence of a low-grade fever. Over the course of ilness, the onset of these symptoms appeared unrelated to her previous cesarean section and was initially attributed to other potential causes, such as dietary issues, irritable bowel syndrome, urinary tract infections, malaria and treated for them accordingly. On examination her vital signs were within normal ranges, and she exhibited mild tenderness on palpation of the RLQ with rest of abdomen soft with a well healed Pfannenstiel wound. Rest of examination was otherwise normal.

Clinical history revealed the patient had undergone a cesarean section two years ago without any immediate postoperative complications. Her surgical recovery had been uneventful, and she had since been leading a healthy life with no significant medical concerns other than the persistent pain and bouts of diarrhea. Laboratory investigations, including a complete blood count and assessment of inflammatory markers, surprisingly yielded results well within the reference ranges. These unremarkable findings initially posed a diagnostic challenge, as the patient's symptoms were indicative of a more insidious underlying issue.

**Fig. 1:**
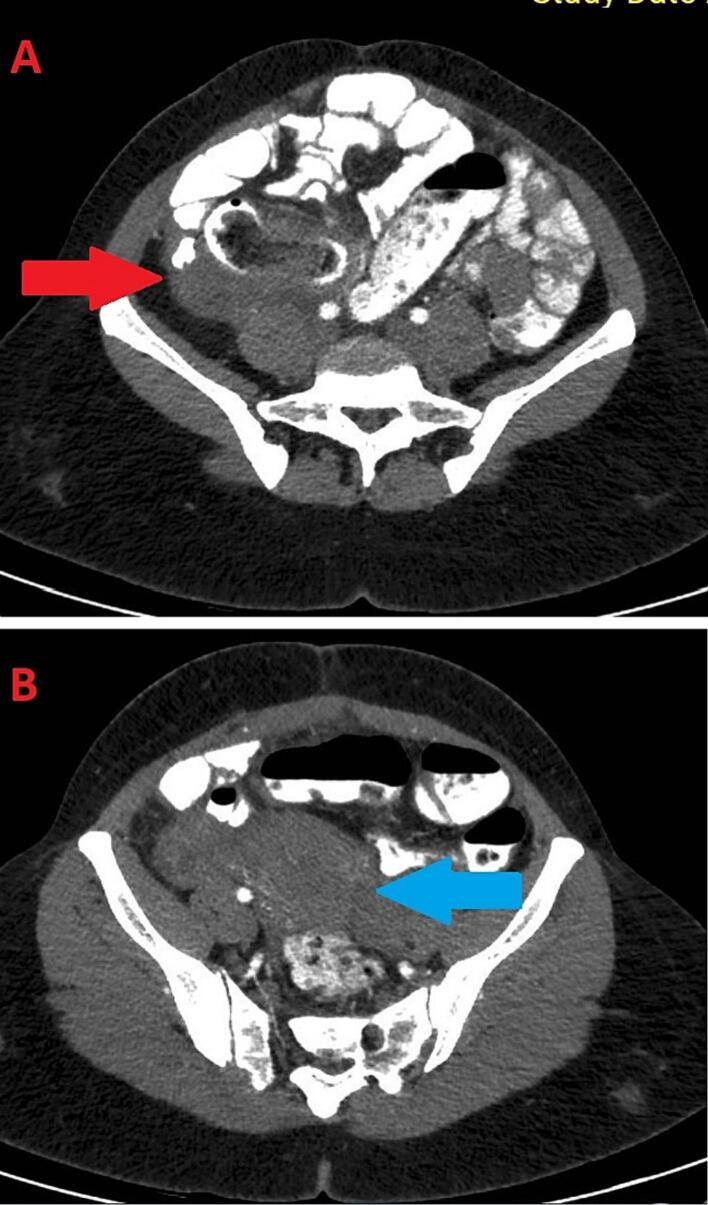
CT scan of abdomen with oral and intravenous contrast axial view. **A** – Showing illdefined iflamatory mass in right illiac fossa involving ileoceacal region with hazziness and streaking with poor plane of demarcation(red arrow). **B** – Showing uterus being pulled towards the right illiac inflamatory mass (blue arrow).

To elucidate the source of her symptoms, a computed tomography (CT) scan of the abdomen and pelvis was promptly conducted (Fig. 1). It revealed an inflammatory mass involving the terminal ileum and caecum with focal thickening and fat stranding with poor plane demarcation. Multiple lymph nodes were seen in the adjacent area and pulling of the uterus towards the inflammatory mass. Of note no obvious foreign body was seen nor suspicion for it on imaging. The decision was made to proceed with a diagnostic laparoscopy to further delineate the mass.

**Fig. 2:**
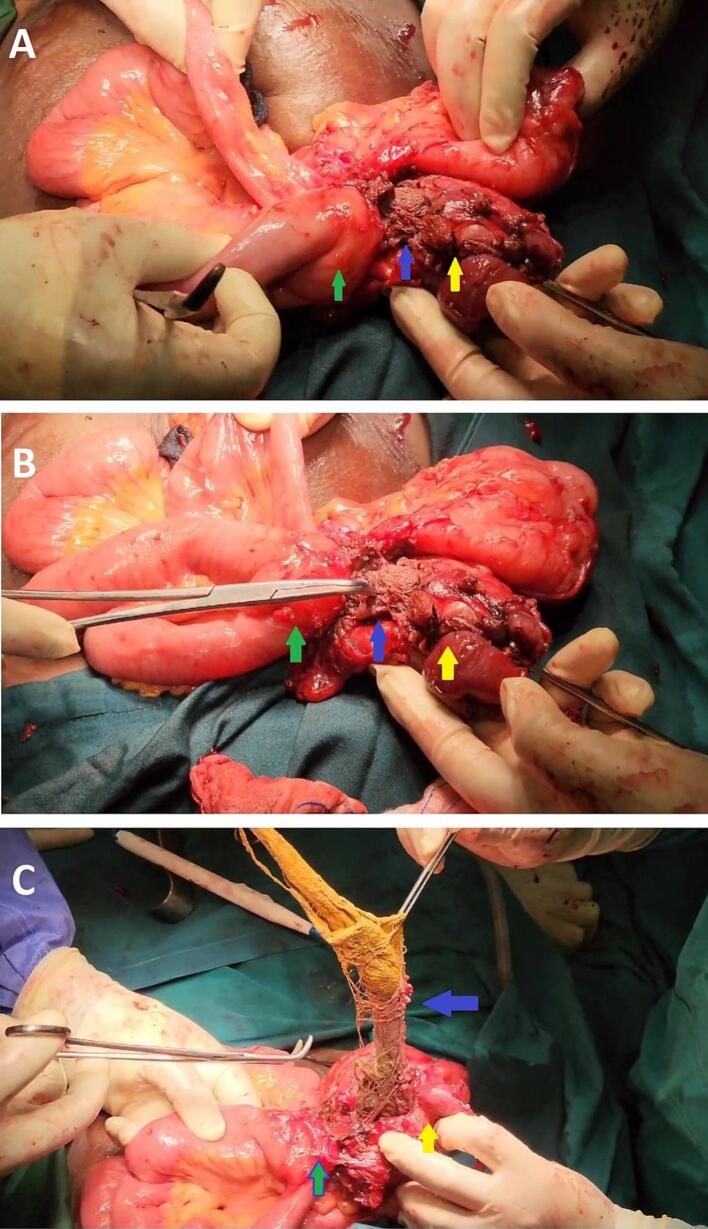
Intraoperative findings of a retained gauze piece (blue arrow) causing fistula between terminal ileum (green arrow) and caecum (yellow arrow). **A** – Identification of fistula upon release of dense adhesions. **B** – Obvious retained gauze seen with invagination into terminal ileum and ceacum. **C** – Removal of gauze piece from the fistula tract within the lumen.

Diagnostic laparoscopy revealed a dense inflammatory mass with difficult planes for dissection to identify anatomy hence the decision to convert to open. Upon release of dense adhesive tissue, a contained abscess with puss fluid was drained and a retained sponge gauze was seen within the lumen of the caecum and terminal ileum causing a fistula formation between the caecum and terminal ileum (Fig. 2). The gauze piece was removed from the lumen of the caecum and terminal ileum and subsequently underwent a right hemicolectomy. The surrounding tissues were inspected for any signs of infection or damage caused by the retained foreign body which only revealed hyperaemia of the right fallopian tube and dome of the uterus which was initially adhered to the inflammation.

Postoperatively, the patient was monitored closely in the surgical recovery unit. She exhibited an uneventful course of recovery, with no immediate complications or signs of infection. She was subsequently transferred to the general surgical ward and received appropriate postoperative care and discharged on day 3 post operative. She is now 3 months post operative and the removal of the retained gauze sponge marked the resolution of the patient's chronic RLQ pain and diarrhea. This surgical intervention not only resolved the patient's long-standing symptoms but also brought attention to the remarkably rare but hazardous occurrence of a retained gauze sponge for an extended duration without the typical signs of infection or abscess formation.

## 3 Discussion

This case of a retained gauze sponge leading to an ileocolic fistula following a cesarean section presents a unique and instructive clinical scenario with several notable discussion points. To the best of my understanding, this is the first case published in our country, Tanzania, on a prolonged foreign body post-gynecological procedure. I believe that most cases are not published due to legal mitigation issues.

The most striking aspect of this case is the atypical presentation of retained foreign bodies [4]. Unlike the classic symptoms of infection and pain seen in most cases, this patient experienced chronic abdominal pain, diarrhea, and low-grade fever over two years [5]. This delayed and subtle presentation challenges clinician to consider the possibility of retained foreign bodies even in cases where symptoms seem unrelated [13].

Diagnostically, this case underlines the importance of advanced imaging techniques. Initial laboratory investigations yielded no conclusive evidence, and the contrast-enhanced CT scan revealed a non-specific inflammatory mass hence needed surgical intervention to truly identify the long standing retained foreign body. Despite advancements in imaging modalities uncovering concealed complications demonstrates a significant difficulty in diagnosing unusual cases [14].

The case also highlights the critical importance of strict adherence to surgical counting protocols. Despite the existence of such protocols, retained foreign bodies continue to occur. This case serves as a reminder that continuous vigilance, robust training, and effective team communication are essential to prevent these incidents. The utilization of radiopaque markers on surgical sponges can further enhance their detectability on imaging studies, potentially aiding in early identification [15].

Patient safety is the central concern in healthcare, and the complications of retained foreign bodies can be severe, leading to additional surgeries, increased costs, prolonged hospital stays, and patient harm [7]. The diligent efforts of the surgical team in promptly identifying and removing the retained gauze sponge demonstrated how early intervention can mitigate the risk of further complications and improve patient outcomes [8,9].

From an educational perspective, this case offers valuable insights for healthcare professionals. It emphasizes the need to consider atypical presentations of retained foreign bodies, especially in patients with chronic and unexplained symptoms. Sharing this case can enhance awareness among healthcare providers and reinforce the significance of established surgical count protocols and the use of radiopaque markers.

Looking to the future, the prevention of retained foreign bodies remains paramount. Healthcare institutions should continually evaluate and update their protocols, invest in comprehensive training and education, and explore innovative technologies to further reduce the risk of such incidents. This case serves as a call to action for hospitals and surgical teams to maintain a culture of safety and prioritize patient well-being in all facets of surgical care.

## 4 Conclusion

In summary, this case report presents a complex and instructive clinical scenario, emphasizing the challenges of diagnosing atypical presentations of retained foreign bodies, the critical importance of surgical counting protocols, and the implications for patient safety and quality of care. It underscores the need for vigilance, education, and preventive measures in surgical practice to ensure optimal patient outcomes.

## Abbreviations

CT: computed tomography
RLQ: right lower quadrant

## Patient's perspective

I am so relieved to be pain free after a long period of time despite going to the hospital multiple times. However, it was disheartening to learn that the cause of all of the pain was due to a mistake of my previous gynaecologist. I am currently pain free and have resumed my daily activity of being a teacher without problems hence am forever grateful.

## Informed consent

Verbal informed consent was obtained from the patient for the anonymized information to be published in this article. Written consent is not required at our institution (Aga Khan University, Tanzania) for case reports if patient particulars are not disclosed in the write up or during use of images.

## Provenance and peer review

Not commissioned, externally peer-reviewed.

## Ethical approval

Ethical approval has been granted from our Aga khan University, with Reference no: AKU: 0176/0 J6/13.

## Funding

This research did not receive any specific grant from funding agencies in the public, commercial, or not-for-profit sectors.

## Author contribution

W.K: Study conception, production of initial manuscript, collection of data, proofreading.

A.I: Revision of the manuscript, proofreading.

S.P: Revision of the manuscript, proofreading.

S.A: Revision of the manuscript, proofreading.

S.S: Production of initial manuscript, collection of data.

A.M: Study conception, production of initial manuscript, collection of data.

## Guarantor

Dr. Ally Mwanga, alimwanga@yahoo.com.

## Research registration number

None.

## Declaration of competing interest

None.
